# Tumeur de Buschke-Lowenstein à localisation pénienne

**DOI:** 10.11604/pamj.2020.37.19.21024

**Published:** 2020-09-05

**Authors:** Ahmed Ibrahimi, Idriss Ziani

**Affiliations:** 1 Service d’Urologie-A, Centre Hospitalo-Universitaire Ibn Sina, Faculté de Médecine et de Pharmacie, Université Mohammed V, 10000, Rabat, Maroc

**Keywords:** Tumeur de Buschke-Lowenstein, condylome acuminé, papillomavirus, pénis, Buschke-Lowenstein tumor, condyloma acuminatum, papillomavirus, penis

## Abstract

Buschke-Lowenstein (BLT) tumor or giant condyloma acuminatum (GCA) is a rare viral disease belonging to the group of verrucous carcinomas; the virus responsible is human papilloma virus (HPV) type 6 and 11 which is transmitted in humans by sexual contact. It is characterized by its extension within the surface layer and at depth, by its degenerative potential and by its recurrent nature after treatment. Therapy is based on surgery. We report the case of a 53-year old patient with a history of recurrent urethritis and a multiplicity of partners without homosexual encounters, presenting with a lesion at the level of the penis. The symptoms started three years before and were caused by a nonpainful budding tumor growing gradually at the level of the root of the penis resulting in tingling and bleeding. Clinical examination showed a dyschromic, infiltrating, cauliflower-like, papillomatous tumor measuring 13cm along its longer axis on the dorsal surface of the penis, with patchy ulcers and extending to the suprapubic region. The remainder of the clinical examination was normal and the assessment of sexually transmitted infection (STI) was negative. Treatment was based on complete surgical resection of the tumor. Soft tissue was covered by the surrounding skin. The anatomopathological study showed a giant genital wart without signs of malignancy. The postoperative course was uneventful. The patient didn’t develop tumor recurrence during a 5-year follow-up period.

## Image en médecine

La tumeur de Buschke-Lowenstein (TBL) ou condylome acuminé géant (CAG) est une affection rare, d’origine virale appartenant au groupe des carcinomes verruqueux. Le virus responsable est le «human papillomavirus (HPV)» de type 6 et 11 qui se transmis chez l’homme principalement par voie sexuelle. Elle se caractérise par son extension en surface et en profondeur, par son potentiel dégénératif et son caractère récidivant après traitement. La chirurgie constitue le moyen thérapeutique de choix. Nous rapportons le cas d’un patient âgé de 53 ans, ayant comme antécédents des urétrites à répétition, et une multiplicité des partenaires sans notion d’homosexualité, consulte pour une lésion au niveau de la verge. Le début de la symptomatologie remontait à trois ans par l’installation progressive d’une tumeur au niveau de racine de la verge non douloureuse bourgeonnante, à l’origine de picotement et de saignement. L’examen clinique trouvait une tumeur dyschromique de 13cm de grand axe, infiltrée, papillomateuse en chou-fleur, ulcérées par endroit, prenant la face dorsale de la verge et s’étendant jusqu’au niveau de la région suspubienne. Le reste de l’examen clinique était sans particularité et le bilan d’infection sexuellement transmissible (IST) était négatif. Le traitement a consisté en une exérèse chirurgicale complète de la tumeur. Le recouvrement cutané a été assuré par la peau de voisinage. L’étude anatomopathologique a montré une lésion en faveur de condylome géant, sans signes de malignités. Les suites opératoires ont été simples. L’évolution avec un recul de 5 ans n’a pas objectivé de récidive tumorale.

**Figure 1:**
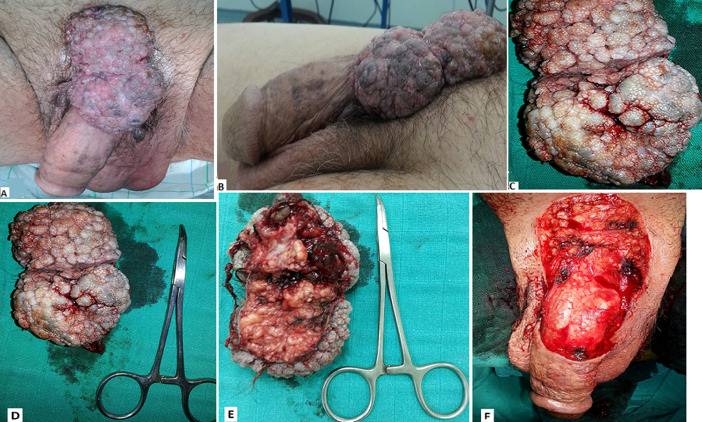
A) aspect clinique de tumeur en chou-fleur avec des lésions bourgeonnantes et végétantes se localisant au niveau de la face dorsale de la verge, la région sus-pubien et l´hemiscrotum gauche (vue de face); B) aspect clinique de tumeur en chou-fleur avec des lésions bourgeonnantes et végétantes se localisant au niveau de la face dorsale de la verge et la région sus-pubien et l´hemiscrotum gauche (vue de profil); C-E) aspect macroscopique de la tumeur après exérèse complète; F) aspect postopératoire après exérèse totale de la tumeur amputant la peau de la moitié proximale de la face dorsale de la verge et s´étendant jusqu´à région sus-pubienne et la partie supérieure de l’hémiscrotum gauche

